# Alignment of Physical Activity in Older Couples Affected by Osteoarthritis: Investigation by Accelerometry and Questionnaire

**DOI:** 10.3390/jcm10071544

**Published:** 2021-04-06

**Authors:** Burkhard Moellenbeck, Frank Horst, Georg Gosheger, Christoph Theil, Leonie Seeber, Tobias Kalisch

**Affiliations:** 1 Department of Orthopedics and Tumor Orthopedics, Muenster University Hospital, Albert-Schweitzer-Campus 1, 48149 Muenster, Germany; burkhard.moellenbeck@ukmuenster.de (B.M.); georg.gosheger@ukmuenster.de (G.G.); christoph.theil@ukmuenster.de (C.T.); leonie-seeber@hotmail.de (L.S.); 2 Department of Orthopedics and Traumatology, St. Josef-Stift Sendenhorst, Westtor 7, 48324 Sendenhorst, Germany; horst@st-josef-stift.de

**Keywords:** osteoarthritis, physical activity, assessment, older couples, behavioral alignment

## Abstract

This study examined whether an alignment of physical activity (PA) between osteoarthritis patients and their spouses, which was previously proven by accelerometry, might also be revealed by self-report. The PA of 28 cohabitating couples (58–83 years) was assessed by means of synchronous accelerometry (ActiGraph wGTX3-BT) and compared to their according self-reports in the German Physical Activity, Exercise, and Sport Questionnaire (BSA-F). Both methods were used to quantify the average weekly light PA, moderate to vigorous PA (MVPA), and total PA. Accelerometry revealed no differences in weekly light PA and total PA (*p* ≥ 0.187) between patients and spouses, whereas the patients’ spouses accumulated significantly more MVPA (*p* = 0.015). In contrast, the self-report did not reveal any differences between the two groups in terms of PA (*p* ≥ 0.572). Subsequent correlation analyses indicated that accelerometry data for mild PA and total PA were significantly correlated in couples (r ≥ 0.385, *p ≤* 0.024), but MVPA was not (r = 0.257, *p* = 0.097). The self-reported PA data, on the other hand, did not indicate any significant correlation (r ≤ 0.046, *p* ≥ 0.409). The presented results give a first indication that an alignment of PA between osteoarthritis patients and their spouses is most likely to be detected by accelerometry, but not by self-report.

## 1. Introduction

Osteoarthritis (OA) of the hip and knee is one of the most common orthopedic conditions that increases with age and is characterized by structural changes of the affected joints, causing pain, functional disability, and a significant loss of autonomy in patients [1,2]. As OA is a progressing degenerative disease, living with OA usually leads to changes in lifestyle that are associated with reduced physical activity (PA) and excessive sedentary behavior [3]. However, regular PA is recommended to reduce pain and maintain or even improve function [2,4]. Unfortunately, patients with hip or knee OA usually do not meet these requirements for PA [5,6].

When the adherence to therapeutic guidelines by patients with chronic diseases is investigated, the influence of the social environment is often ignored. Most PA studies primarily focus on the affected patients, neglecting the role of their partners [7], although PA is strongly influenced by the people around us [8]. In particular, individuals in permanent relationships share their daily environment, engage in mutual activities, and care about each other’s health. Therefore, a patient’s relationship and partners must be considered when studying PA [8]. In this context, it has been shown that moderate to vigorous PA (MVPA) and sedentary behavior (i.e., sedentary time) are correlated in couples [9,10,11]. In addition, longitudinal studies revealed that when one partner changes their activity level, it is more likely that the other partner will change their behavior in a similar way [12,13]. Social theories suggest that there may be two distinct underlying behavioral mechanisms that also have implications for intervention development [14]. First, behavioral concordance may be a result of life circumstances shared by couples (everyday life, social and financial aspects, etc.) that affect both partners in similar ways [15]. Second, partners may also influence each other’s behavior in beneficial or detrimental ways [16]. However, this theoretical background has only sparsely been studied in concrete experimental settings [17].

Nevertheless, for patients who are in a relationship, the partner is the primary coping resource [18]. Particularly in OA patients, it has been shown that it might be beneficial to include partners in couple-oriented interventions that are related to PA [9], as it could be shown that the PA of couples synchronizes and aligns over time [17]. While this alignment is of minor importance in healthy couples, it might lead to maladaptive behavior in the spouses of patients with musculoskeletal diseases and restricted mobility [19].

There are a variety of methods to measure PA in daily life. Probably the most appropriate method is the use of accelerometers [20], as they can be used to implement an activity event-based approach, and thus objectively capture the F.I.T.T. (i.e., frequency, intensity, time, and type of the PA) dimensions of PA, depending on their technical design [21]. In addition, these devices can objectively and reliably determine the amount of time spent sedentary, which is known to affect the overall health and functional status of OA patients [22]. Although accelerometry has become the standard for the objective assessment of PA and sedentary behavior in various populations, questionnaires are widely used, considering easy availability, rapid application, and low costs [23]. Nevertheless, it has been shown that PA information derived from self-reports is potentially prone to response bias [23,24,25]. Correlations between self-reported and objective measures in patients were reported as mostly weak to moderate. Measurement methods and sociodemographic and health factors were associated with the observed differences in reporting PA among patients with OA [26].

The intention of the present study was to examine whether questionnaire data on PA collected from older couples consisting of OA patients and their spouses also show a correlative association. Despite the already-known discrepancies between self-reports and objective measurements, this could be the case if both partners equally over- or underestimate their own PA.

## 2. Materials and Methods

The presented data were obtained in a cross-sectional, exploratory study conducted to assess PA by means of accelerometry and self-report in a sample of German end-stage OA patients and their cohabitating partners. Since all partners were married to the patients, the term spouse is used.

### 2.1. Sample Recruitment and Data Collection

The criteria for inclusion of patients were symptomatic hip or knee OA (according to the guidelines of the American College of Rheumatology [27]), age between 50 and 85 years, and sufficient language skills in German to understand the requirements of the study and answer the questionnaires adequately. Exclusion criteria were defined as any non-orthopedic condition significantly limiting PA in everyday life or enforcing a sedentary lifestyle. The spouses lived together with the patients in one household on a permanent basis and had to be without any medical history of knee or hip OA (i.e., current treatment status or medication). As for the patients, the exclusion criteria were defined as any disease or injury seriously limiting PA in everyday life or enforcing a sedentary lifestyle.

Self-reported characteristics that were associated with levels of PA in the published literature, including age, body mass index, gender, and education [28], were considered. Comorbidities were assessed by asking the study participants if they were currently receiving regular medical treatment and/or receiving medications for any of the following conditions: cardiovascular, pulmonary, metabolic, gastrointestinal, liver, kidney, blood, cancer, depression, or musculoskeletal diseases. Their responses were recorded as a score (0–10).

As datasets were selected from a larger collective, which had already been reported in a previous study, details on the recruitment of the volunteers can be found elsewhere [19]. Data of 28 patients (11 males and 17 females; 71.11 ± 5.47 years; range 61–81 years) and their 28 spouses (17 males and 11 females; 70.82 ± 7.29 years; range 50–83 years) were analyzed. All patients met the clinical and radiological criteria for hip or knee OA and were scheduled for elective arthroplasty.

### 2.2. Instruments

Accelerometry. PA was assessed by tri-axial ActiGraph wGTX3-BT (Firmware 1.9.2) activity monitors (ActiGraph LLC, Fort Walton Beach, FL, USA) that were worn on the waist using elastic belts. The couples were instructed to wear the monitors simultaneously and remove them only for water-based activities, such as bathing and swimming. The monitors were initialized per the manufacturer’s manual. Data were downloaded using the ActiLife Software (ver. 6.13.3, ActiGraph LLC, Fort Walton Beach, FL, USA) with normal filter settings. The minimum wear time was set to 10 h per day for at least four days, as this number is minimally needed to obtain reliable PA estimates [29]. Furthermore, at least one day of the weekend was included, as a higher activity level is generally expected here [30]. Night hours (11:01 p.m.–05:59 a.m.) were excluded from the data acquisition by default, as the participants typically slept during this time. The monitors’ sampling frequency was set at 100 Hz, and the epoch length at 10s. Non-wear times were automatically detected and excluded from analyses based on the Choi algorithm [31]. Analyses were based on cut-points (for uniaxial sensor data) by Matthew et al. [32] for sedentary behavior (0–99 CPM), light PA (100–759 CPM), MVPA (≥760 CPM), and total PA (≥100 CPM). The total duration of PA in each intensity range was recorded without a minimum duration (i.e., bout). The data collection on the couples was carried out in a comparatively short period of time (end of May to mid-July), so that bias due to seasonal or environmental factors (e.g., outdoor exercise, sports, cultural activities, etc.) was kept to a minimum. The accuracy and test–retest reliability of ActiGraph accelerometers under field conditions are established in many populations, including persons with OA [33].

Patient-reported outcome measure. The Lequesne Index (LI) covers specific symptoms and physical functional disability in patients with hip or knee OA [34]. It aggregates symptoms and function, where pain is analyzed by five items, maximum distance walked by two items and activities of daily living by four items. The score ranges from 0 (no pain/disability) to 24 (maximum pain/disability), and is scored as the sum of all questions, where 0 is no disability, 1–4 equals a mild disability, 5–7 equals a moderate disability, 8–10 equals a severe disability, 11–13 equals a very severe disability, and 14 equals an extremely severe disability [35]. The test quantifies the general self-perception of the patient with regard to his state of health. The LI was used at the time of patient recruitment.

Questionnaire. The individual self-assessment of everyday PA (corresponds to light PA) and sport activities (corresponds to MVPA) in OA patients and spouses was conducted using the German Physical Activity, Exercise, and Sport Questionnaire (BSA-F: derived from German: Bewegungs- und Sportaktivität Fragebogen), which was designed to address the F.I.T.T. (frequency, intensity, time, and type of the PA) dimensions of PA. By means of the BSA-F basic PA (i.e., all physical movements that provoke a substantial increase in energy consumption through the use of larger muscle groups) in everyday life, at work, and during leisure time are quantified by nine items (e.g., walking, cycling, gardening, housework, caring for or supporting relatives). In addition, individual sport activities (not only competitive sports, but also fitness and exercises) are assessed. For this purpose, the frequency and duration of every specific activity during the last four weeks is queried and reported as cumulative indices (i.e., average minutes per week) for PA (here referred to as the everyday activity index) and sports (the sport and exercise index) [36]. In view of the fact that the majority of the study participants had already retired (cf. Table 1), the separate index on PA at work was not used. In this study, both indices were summed and reported as a total activity index. The period of four weeks analyzed by the BSA-F questionnaire included the time during which the couples’ PA was measured by means of accelerometers.

### 2.3. Statistical Analysis

All subject data were pseudonymized. Results were presented as means (M) ± standard deviation (SD) or as median (Mdn) and interquartile range (IQR). Data were checked on a normal distribution by means of the Shapiro–Wilk test. In the case of a violation of the normality assumption, non-parametric methods were used. Either *t*-tests, chi-square tests, or Mann–Whitney U-tests were used to examine differences between the patients and their spouses. The correlation of data obtained by self-report and accelerometry was investigated with Pearson or Spearman rank correlations per group (i.e., patients or spouses). The correlation of data obtained by the same test in both groups was investigated by partial correlations correcting for the difference in the BMI between the partners. An a priori sample size calculation for correlation analyses indicated that a minimum sample size of *N* = 19 subjects was required to achieve a significant strong (r = 0.6 according to the working hypothesis) correlation between data quantifying the patients’ PA and their spouses’ data with a statistical power of 0.80 and an error probability of 0.05. All statistical analyses were performed using SPSS (v26; SPSS Inc., Chicago, IL, USA), with the significance level set at α = 0.05.

## 3. Results

### 3.1. Clinical and Sociodemographic Characteristics of the Study Population

All 56 participants (*N* = 28 OA patients, *N* = 28 spouses) who were selected for the study provided data. The sociodemographic characteristics of the subjects are summarized in Table 1.

On average, the patients were diagnosed with OA 7.2 ± 6.7 years ago (first medical treatment because of joint discomfort). The initial degree of OA-related restrictions under everyday conditions was determined by the according LI for the hip or knee [34]. There was no significant difference between patients with knee (LI_knee_: 11.63 ± 3.13) and hip osteoarthritis (LI_hip_: 10.00 ± 2.80) (*p* = 0.161).

In total, accelerometric data from OA patients and their spouses were recorded in 5.98 ± 0.84 days. There was no significant difference between the valid days collected (*p* = 0.638), and the duration of measurement was significantly correlated in both groups (r = 0.934, *p* < 0.001). Data obtained by accelerometry were approximately normally distributed (patients: *p* ≥ 0.381; spouses: *p* ≥ 0.064), whereas data of the BSA-F questionnaire were not normally distributed (patients: *p* ≤ 0.01; spouses: *p* ≤ 0.01).

Accelerometry revealed no significant differences in the sedentary behavior, light PA, and total PA of the patients and their spouses (*p* ≥ 0.187). Cumulative MVPA, on the other hand, was significantly higher among the spouses by 200 min per week (*p* = 0.015). The data collected by the BSA-F questionnaire did not reveal any differences between the patients and their spouses, neither for the everyday activity index, the sport and exercise index, nor for the total activity index (*p* ≥ 0.572) (Table 2).

#### 3.1.1. Group-Specific Interrelatedness of PA Assessments

Correlation analyses were carried out to investigate the interrelationship of the investigated parameters within and between the two used instruments. First, it could be shown that most parameters of accelerometry (sedentary time, time in light PA, MVPA, and total PA) were significantly correlated, both for the patients’ data (r ≥ −0.478, *p ≤* 0.005) and their spouses’ data (r ≥ −0.433, *p ≤* 0.011). Only the correlation between the patients’ sedentary time and their light PA failed to meet the significance criteria (r = −0.169, *p* = 0.196). Contrasting that, the everyday activity indices and the sport and exercise indices of the questionnaire were not correlated, neither in patients nor in their spouses (r ≤ 0.157, *p* ≥ 0.213). However, both indices were correlated with the total activity index (r ≥ 0.381, *p ≤* 0.023).

Subsequent analyses investigating the interrelationship between the accelerometry data and the questionnaire data revealed that the everyday activity indices of the questionnaire were significantly correlated with all parameters of accelerometry for both patients (r ≥ −0.332, *p ≤* 0.042) and their spouses (r ≥ 0.444, *p ≤* 0.009). Sport and exercise indices, on the other hand, were not correlated with any accelerometry parameter, neither for the patients’ data nor for their spouses’ data (r ≤ −0.307, *p* ≥ 0.056) (Table 3).

#### 3.1.2. Alignment of PA Between the OA Patients and Their Spouses

**Figure 1:**
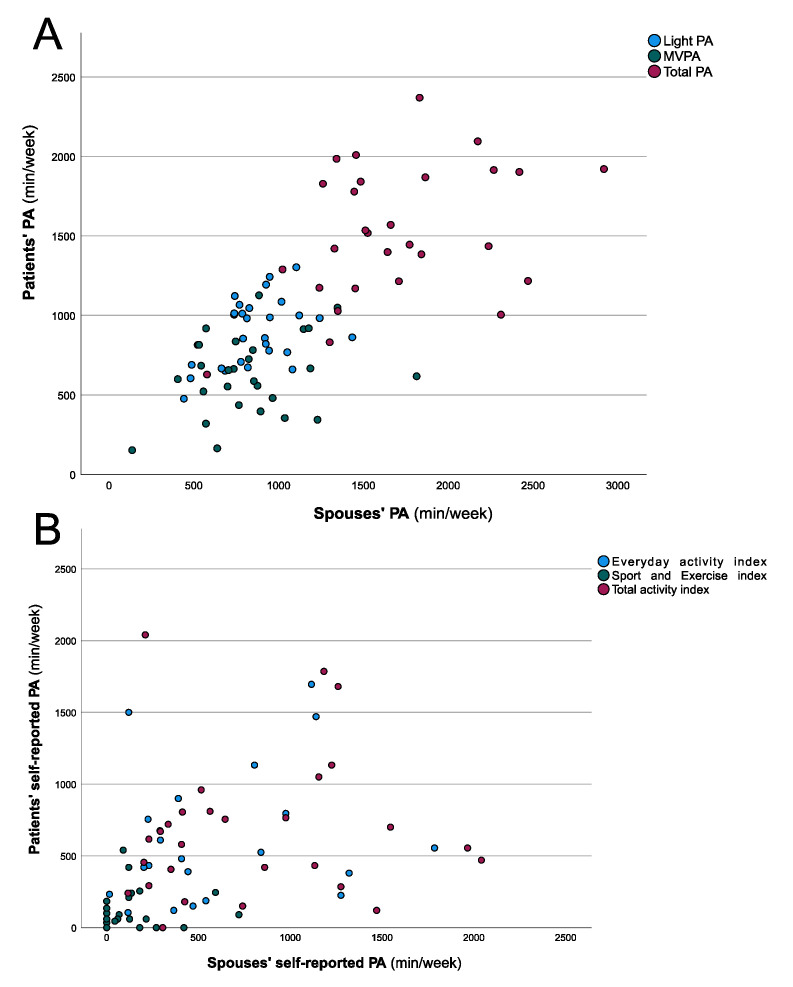
Correlation analyses of the patients’ and their spouses’ PA. (**A**) Scatterplot of the participants’ PA based on accelerometer data. The partial correlations (controlled for the couple-specific deviations in BMI) of weekly light PA and total PA revealed significant results (r ≥ 0.331, *p* ≤ 0.046), whereas MVPA showed no significant results (r = 0.257, *p* = 0.097). (**B**) Scatterplot of the participants’ weekly PA based on self-reported data. The partial correlations of everyday activity, sport, and exercise and total activity indices showed no significant results (r ≤ 0.046, *p* ≥ 0.409).

To compensate for couple-specific deviations in BMI that might influence PA, partial correlation methods were used, in which the influence of the BMI is kept constant. Three correlation analyses between the patients’ and their spouses’ accelerometer parameters showed significant results. There was a significant correlation between the patients’ sedentary behavior and their spouses’ sedentary behavior (r = 0.331, *p* = 0.046), between their weekly time spent in light PA (r = 0.410, *p* = 0.017), and their weekly total PA (r = 0.385, *p* = 0.024). Only the time in MVPA per week was not correlated between the patients and their spouses (r = 0.257, *p* = 0.097) (Figure 1A). On the other hand, there were no significant correlations found between the patients’ and their spouses’ self-reported PA. Neither the everyday activity indices (r = 0.046, *p* = 0.409), the sport and exercise indices (r = 0.041, *p* = 0.419), nor the total activity indices of the questionnaire (r = −0.046, *p* = 0.410) reached significance criteria (Figure 1B).

## 4. Discussion

In this study, we described the levels of weekly PA using both self-reported and objectively measured PA among middle-aged and elderly German couples affected by OA. Previous studies have shown that there is an alignment of health-related behavior between healthy couples in particular with regard to PA and sedentary behavior [17]. Reduced PA, as inevitably occurs in the context of musculoskeletal diseases, consequently poses an increased risk for morbidity and mortality not only to patients, but also to their partners—an aspect that has received little attention so far. OA patients, whose daily lives are typically characterized by long periods of sitting [37], short walking distances, and rare MVPA [6], should be highlighted in this context. Using accelerometry, it has already been shown that the couple-specific deviation in everyday PA is significantly smaller in couples consisting of OA patients and their spouses than in control couples [19]. Here, we investigate the methodological question of whether an alignment of PA between OA patients and their spouses could also be revealed by self-report.

### 4.1. PA in OA Patients and Their Spouses under Everyday Conditions

Examining the correlation between self-reported and objectively measured PA is particularly important in individuals with OA, because they may have a different lifestyle and are often recommended to engage in specific activities, such as low-impact aerobic exercise [38]. The OA patients included in this study spent more than two-thirds of their waking time sedentary, which is in agreement with data known from the literature [22]. The same applied to their cohabitating spouses. The agreement of the cumulative sedentary times of the two groups is remarkable. Although these similarities suggest a highly synchronized lifestyle, behavioral alignment of sedentary behavior and indices of PA must nevertheless be investigated independently.

Both methods showed broad agreement in that no differences were seen between the investigated groups in terms of weekly light PA and total PA. However, a comparison of the number of active minutes per week recorded by the two methods showed massive differences. The light PA determined by accelerometer was about twice as high as the self-reported everyday activity index, and the total PA was about three times as high as the total activity index. These differences could be explained by known problems with PA questionnaires in general, as self-reported levels of PA are based on a persons’ perception of his or her own quantity of PA, and are therefore prone to misinterpretation and social desirability, and are reliant on accurate recall of the type, intensity, frequency, and duration of activities [39]. On the other hand, there were differences in the time frame of data collection, as the BSA-F questionnaire covered the last four weeks, and the accelerometers only covered the last four to seven days.

The investigation of PA often focuses on MVPA because it is the recommended intensity to meet national physical activity guidelines for aerobic activity [40]. MVPA is defined as any activity ≥3 metabolic equivalents of a task, and therefore is not limited to exercise. It can be performed as part of daily life activities, such as lawn raking, vacuuming, or brisk walking [41]. This explains the extreme difference between the MVPA times recorded by questionnaire and accelerometer in this study. It can be assumed that a large part of the objectively recorded MVPA times were recalled by the study participants as everyday activities in the questionnaire. As a bout criterion (i.e., an uninterrupted minimum period of a specific activity) was not considered in the present study, the generally recommended 150 min of MVPA per week [41] is easily reached by both groups in our study. It should be noted that the latest U.S. physical activity guidelines highlighted the benefits of light activity and the importance of including all activities across the day, regardless of bout lengths [42]. The higher MVPA of the spouses in the present study might also be explained by some kind of compensatory takeover of daily activities that the patients are unable to perform (housekeeping, shopping, etc.) [43].

### 4.2. Correlation of PA Assessed by Different Methods

When analyzing the PA data collected by means of the two methods, corresponding correlations were found within the two groups of subjects. In both groups, light PA was correlated with the everyday activity index, and total PA was correlated with the total activity index, suggesting a good fit between the two methods in determining PA in these activity domains. The range of correlation within the spouses’ data was weak to moderate, which is similar to previous findings in healthy adults [44,45,46]. MVPA, on the other hand, was consistently not correlated with the sport and exercise index. This could be due to a weakness in the BSA-F questionnaire used. In contrast to the everyday activity index, the subjects were not given any exemplary activities for the sport index, but had to name up to three practiced sporting activities. Against the background of the drastically lower sport indices compared to the accelerometer data, many subjects seem not to have perceived their own PA as sportive. It should be investigated whether this discrepancy also occurs with a survey instrument that specifically asks about activities that are typical for older adults. It is known that older adults are more likely to engage in light- and moderate-intensity PA, which is the most difficult type of activity to be assessed by self-report [47,48]. Current studies investigating the alignment of PA in couples utilize the full potential of accelerometry by calculating hour-by-hour covariation [17]. Pauly and colleagues revealed that MVPA and sedentary synchrony were higher in the morning and evening and more pronounced on weekends [17]. However, in the present study, the MVPA parameter studied was a weekly mean that could be directly compared to the self-reported data. Generally, the correlation between survey data and accelerometer data is greater in younger people than in older people who participated in our study [49]. Overestimating or underestimating one’s activity is a known problem when answering PA questionnaires [26,50]. OA patients might misclassify their own activity levels and the times spent on certain activities due to chronic pain conditions and the resulting frequent interruptions of light PA and especially MVPA. Van Weering and co-workers showed that questionnaire and accelerometer data of participants in a control group who were not affected by a chronic pain condition were correlated strongly [51].

However, the main aspect of this study was the comparative investigation of the PA alignment between OA patients and their spouses using objective and subjective methods. Both methods were aimed at assessing light PA, MVPA, and total PA. Accelerometry showed that at least two of the three parameters were significantly correlated between the partners, whereas self-reported data revealed no significant correlations. In addition, sedentary behavior quantified by accelerometry was also correlated between the partners, but, unfortunately, the corresponding assessment was missing in the used questionnaire. On the one hand, these findings largely confirm previous results regarding the behavioral alignment between couples in general [17] and between OA patients and their spouses in particular [19]. On the other hand, we provide a first indication that a behavioral alignment between OA patients and their partners may not be detected by survey instruments alone.

The relatively low correlation and large differences between accelerometer-measured and self-reported data observed in this study, as well as in others, is not surprising given that they do not measure the same constructs [52]. Questionnaires capture the perceived time spent in specific activities, while sensors capture movement or continuous measures of bodily acceleration above a defined threshold [53]. Leading experts assert that direct comparisons between self-reported and measured PA are unsuitable [53], and that these methods should be better used in a complementary way [54].

### 4.3. Strengths and Limitations of the Study

To our knowledge, this study is the first to investigate the differences between accelerometric and questionnaire-based assessment of PA alignment in OA patients and their spouses. The synchronous measurements, which lasted four to seven days, fulfilled the requirements for an objective recording of habitual PA using accelerometry [55]. The data collection with both methods coincided in time to promote the comparability of data.

Some limitations of this study must be acknowledged. There are aspects that limit PA measurement by accelerometry, such as the devices not being able to cover stationary activities, cycling, or water-based activities [46]. In addition, the wearing of an accelerometer itself may promote PA [56]. The relatively small population of patients in this study is not a homogeneous one with respect to OA, as patients with OA of the hip and knee were included. Although the degree of OA-related limitation is comparable in both groups, it is known that patients with severe OA of the knee are less likely to meet PA guidelines compared to hip OA patients [6]. In further studies, the proportion of time spent on sedentary activities should also be determined by self-report in order to compare it with the accelerometer data. Unfortunately, this was not done in the current study.

## 5. Conclusions

Self-reported and accelerometer-measured PA differs largely in couples with one partner suffering from OA. While self-reported everyday activity, sport and exercise activity, as well as total activity were uncorrelated between patients and spouses, significant correlations of the couples’ light PA and total PA were demonstrated using accelerometry. Self-reported PA does not seem to be suitable to study behavioral alignment in this population.

## Figures and Tables

**Table 1:** Demographic characteristics of the OA patients and their spouses.

Variable	OA Patients (*n* or M ± SD)	Spouses (*n* or M ± SD)	Difference between Groups	Couple-Specific Deviation (M ± SD)
Age	71.11 ± 5.47 (range 61–81 years)	70.82 ± 7.29 (range 58–83 years)	*p* = 0.869	2.93 ± 2.11
Gender	11 male, 17 female	17 male, 11 female	χ^2^ = 2.571; *p* = 0.109	-
BMI ^a^	23.03 ± 3.72	26.98 ± 3.95	*p* = 0.964	3.08 ± 2.90
Education ^b^	7 sl1, 15 sl2, 3 tl ^§^	11 sl1, 12 sl2, 3 tl ^§^	χ^2^ = 1.203; *p* = 0.548	0.46 ± 0.58
Professional status ^c^	24 r, 2 pt, 2 ft	24 r, 2 pt, 2 ft	χ^2^ = 0; *p* = 1	0.14 ± 0.45
Comorbidities ^d^	1.29 ± 0.76	0.93 ± 0.81	*p* = 0.096	0.79 ± 0.74
Pain medication ^e^	7 n, 11 i, 2 w, 3 ww, 5 d	-	-	-

^a^ Body mass index (body mass divided by the square of the body height (kg/m^2^)). ^b^ Education levels: pl (primary level; age: 6–10 years), sl1 (secondary level I; age: 10–15/16 years), sl2 (secondary level; age: 15–19 years), and tl (tertiary level; age: >19 years) (0 = lowest education; 4 = highest education); ^§^ data missing. ^c^ Professional status (r = retired; pt = part-time job; ft = full-time job). ^d^ Pathological conditions (0 = best condition; 10 = worst condition). ^e^ Analgesic consumption related to osteoarthritis (n = none | i = irregular | w = weekly | ww = several times a week | d = daily).

**Table 2:** Physical activity of OA patients and their spouses.

Test	OA Patients	Spouses	Difference in Activity
Accelerometry (min/week)	M ± SD		*t*-test
Sedentary Behavior	4190.06 ± 585.85	4178.97 ± 657.15	*p* = 0.947
Light PA	897.20 ± 209.32	864.31 ± 223.46	*p* = 0.572
MVPA	630.72 ± 247.80	828.47 ± 335.43	*p* = 0.015 *
Total PA	1527.93 ± 415.93	1692.78 ± 503.33	*p* = 0.187
Self-report [min/week]	Mdn (IQR)		Mann–Whitney test
Everyday activity index	456.25 (226.88–785.00)	427.50 (290.63–1080.00)	U = 371.00, Z = −0.344, *p* = 0.731
Sport and exercise index	60.00 (0.0–171.56)	63.75 (0.0–180.0)	U = 382.50, Z = −0.161, *p* = 0.872
Total activity index	598.13 (320.88–808.75)	603.75 (312.50–1214.38)	U = 357.5, Z = −0.565, *p* = 0.572

* significant result (*p* ≤ 0.05).

**Table 3:** Group-specific interrelatedness of PA determination by accelerometry and self-report.

Patients’ PA	Light PA/ Everyday Activity Index	MVPA/ Sport and Exercise Index	Total PA/ Total Activity Index
Sedentary behavior	ACC: r = −0.169, *p* = 0.196 X: r = −0.332, *p* = 0.042 *	ACC: r = −0.660, *p* < 0.001 * X: r = 0.006, *p* = 0.489	ACC: r = −0.478, *p* = 0.005 * X: r = −0.361, *p* = 0.030 *
Light PA/ Everyday activity index	X: r = 0.410, *p* = 0.015 *	ACC: r = 0.653, *p* < 0.001 * QST: r = 0.121, *p* = 0.270 X: r = −0.058, *p* = 0.384	ACC: r = 0.893, *p* < 0.001 * QST: r = 0.925, *p* < 0.001 * X: r = 0.315, *p* = 0.051
MVPA/ Sport and exercise index	X: r = 0.367, *p* = 0.027 *	X: r = −0.053, *p* = 0.395	ACC: r = 0.925, *p* < 0.001 * QST: r = 0.396, *p* < 0.018 * X: r = 0.307, *p* = 0.056
Total PA/ Total activity index	X: r = 0.430, *p* = 0.011 *	X: r = −0.092, *p* = 0.321	X: r = 0.338, *p* = 0.039 *
**Spouses’ PA**	**Light PA/** **Everyday activity index**	**MVPA/** **Sport and exercise index**	**Total PA/** **Total activity index**
Sedentary behavior	ACC: r = −0.433, *p* = 0.011 * X: r = −0.490, *p* = 0.004 *	ACC: r = −0.670, *p* < 0.001 * X: r = −0.307, *p* = 0.056	ACC: r = −0.638, *p* < 0.001 * X: r = −0.609, *p* = 0.001 *
Light PA/ Everyday activity index	X: r = 0.567, *p* < 0.001 *	ACC: r = 0.606, *p* < 0.001 * QST: r = 0.157, *p* = 0.213 X: r = 0.148, *p* = 0.227	ACC: r = 0.884, *p* < 0.001 * QST: r = 0.947, *p* < 0.001 * X: r = 0.572, *p* < 0.001
MVPA/ Sport and exercise index	X: r = 0.444, *p* = 0.009 *	X: r = 0.175, *p* = 0.186	ACC: r = 0.936, *p* < 0.001 * QST: r = 0.381, *p* < 0.023 * X: r = 0.537, *p* = 0.002 *
Total PA/ Total activity index	X: r = 0.555, *p* < 0.001 *	X: r = −0.119, *p* = 0.274	X: r = 0.601, *p* < 0.001 *

Group-specific correlation analyses were carried out with accelerometry data (ACC: sedentary behavior, light PA, MVPA, and total PA), on data of the BSA-F questionnaire (QST: everyday activity index, sport and exercise index, and total activity index), and on data across methods (X). Sedentary behavior was detected exclusively by means of accelerometry. * Significant result (*p* ≤ 0.05).

## Data Availability

The datasets generated and analyzed during the current study are not publicly available but are available from the corresponding author upon reasonable request.
